# An improved PBFT consensus algorithm based on grouping and credit grading

**DOI:** 10.1038/s41598-023-28856-x

**Published:** 2023-08-10

**Authors:** Shannan Liu, Ronghua Zhang, Changzheng Liu, Chenxi Xu, Jiaojiao Wang

**Affiliations:** 1 College of Information Science and Technology, Shihezi University, Shihezi, 832000 Xinjiang China; 2 School of Economics and Management, Qilu Normal University, Jinan, 250200 Shandong China

**Keywords:** Computer science, Information technology

## Abstract

To improve the blockchain consensus algorithm practical Byzantine fault tolerance (PBFT) with random master node selection, which has high communication overhead and a small supported network size, this paper proposes a Byzantine fault tolerant consensus algorithm based on credit (CBFT) enhanced with a grouping and credit model. The CBFT algorithm divides the network nodes according to the speed of their response to the management nodes, resulting in different consensus sets, and achieves consensus within and outside the group separately to reduce communication overhead and increase system security. Second, the nodes are divided into different types according to the credit model, each with different responsibilities to reduce the probability that the master node is a malicious node. Experimental results show that the throughput of the CBFT algorithm is 3.1 times that of PBFT and 1.5 times that of GPBFT when the number of nodes is 52. Our scheme has latency that is 7.4% that of PBFT and 38.8% that of GPBFT; CBFT has communication overhead that is 6.4% that of PBFT and 87.3% that of GPBFT. The number of nodes is 300, and the Byzantine fault tolerance is improved by 59.3%. These improvements are clearer with the increase in the number of nodes.

## Introduction

Blockchain technology originated from Bitcoin and was proposed by an academic named Satoshi Nakamoto in 2008^1^. It is essentially a distributed database that integrates distributed computing, cryptography, network transmission and other technologies and is decentralized, tamper-proof, programmable and traceable^2–4^.

The research on consensus algorithms started before the emergence of blockchain technology, and as the core of blockchain, it is mainly responsible for the correctness and consistency of data transmission and processing^5^. That is, the same ledger information is kept on the local nodes to avoid data tampering, and the data are quickly detected during the consistency protocol to ensure data security effectively^6^. Consensus algorithms can be classified into two categories, crash fault tolerance (CFT) and Byzantine fault tolerance (BFT), based on whether the system can solve the Byzantine errors^7^. CFT consensus algorithms cannot handle Byzantine errors and are mainly used in private chains^8^; BFT consensus algorithms can handle Byzantine errors and are widely used in public and consortium chains^9^; ^10^. The consensus algorithms of the BFT class are proof of work (PoW)^11^, proof of stake (PoS)^10^, delegated proof of sequiency (DPoS)^12^ and practical Byzantine fault tolerance (PBFT). PoW mainly operates on SHA-256 cryptographic hash functions by computer arithmetic, which is not only computationally intensive but also less efficient in terms of consensus. The PoS algorithm emerged as an attempt to address the large amount of resources being wasted in the POW mechanism by calculating the percentage of holdings to the total number of coins and the time to hold the coins to determine the bookkeeping rights. The algorithm overcomes the disadvantages of large amounts of arithmetic power to a certain extent but struggles with an uneven distribution of resources, coin holdings that tend to be centralized and less liquid, etc. The DPoS mechanism is optimized on the basis of PoS, where nodes elect producers to fulfill their rights and obligations on their behalf through voting elections, improving the efficiency of block generation but also deviating from the principle of decentralization. The above algorithms can ensure the reliability of the blockchain to a certain extent, but they all have their own shortcomings and cannot solve the problems of latency, throughput and security at the same time.

The PBFT consensus algorithm was improved by Castro et al.^13^ based on the BFT algorithm, which is used to solve the problem of large computation of the BFT algorithm, reducing the complexity of the algorithm from the exponential to the polynomial level, and still enables distributed nodes to reach consensus in the presence of a certain number of Byzantine nodes, which is recognized as the optimal algorithm to solve the Byzantine general problem^14^^,^^15^. However, the PBFT algorithm is degraded by the problems of the arbitrary master node selection method, high complexity of three-stage protocol communication, and the inability of nodes to join and exit dynamically, which reduces the performance of the consortium chain^16^.

To solve the above problems, this paper proposes an improved PBFT consensus algorithm that is more efficient and secure. The scheme can ensure low communication overhead and high fault tolerance of the consortium chain. The main contributions of this paper are as follows.

- A node grouping model is proposed. Based on the response speed of the nodes of the consortium chain to the management nodes as a basis for grouping, the in-group consensus is performed first, and the management nodes take the result of the in-group consensus and then participate in the out-group consensus.
- A credit mechanism is proposed. By introducing a credit calculation formula, the nodes in the group are divided into management nodes, candidate nodes, and common nodes, and the nodes with high credit are selected from the candidate nodes as management nodes. The common nodes do not participate in consensus but accept the consensus results, which can improve the consensus efficiency of large consortium chains.
- A simulation and performance testing system based on this improved scheme is built. The effectiveness, as well as usability of the scheme, is demonstrated through simulation experiments. The experimental results show that the CBFT consensus algorithm has smaller latency, higher throughput, and less communication overhead, which can support larger-scale traceability, effectively improve the security of the system and reduce the impact of malicious nodes.

The rest of the paper is organized as follows. Section 2 describes the current state of the research on the improved PBFT algorithm. Section 3 discusses the working principles and flaws of PBFT. Section 4 provides a methodological description of the CBFT algorithm, including the grouping strategy and credit model. In Sect. 5, the implementation of the CBFT algorithm is demonstrated through simulation experiments. Section 6 concludes the paper.

## Related work

The Byzantine fault tolerance (BFT) algorithm, proposed by Pease and Lamport in 1980^17^^,^^18^ analyses the way to achieve data consistency and integrity in peer-to-peer networks with malicious nodes or network blockage problems. The PBFT consensus algorithm was improved by Castro et al.^13^ based on the BFT algorithm for solving the consensus problem of distributed systems in current consortium chains. The PBFT algorithm inherits the advantages of BFT that can tolerate Byzantine nodes and reduces the communication complexity from O(n^3^) to O(n^2^) in the BFT algorithm with a relatively high consensus efficiency, a small number of starting nodes and a fault tolerance rate close to 1/3. It is a more widely used consensus algorithm in consortium chains^19^^,^^20^. However, the PBFT consensus algorithm has shortcomings in some aspects^21^. First, the selection of master nodes in the PBFT algorithm is based on the rotation of master nodes by number, which is vulnerable to DDoS attacks^22^ and sybil attacks^23^ in P2P networks and has security risks. Second, the broadcasting process of the PBFT three-stage protocol requires network-wide forwarding with great communication overhead, which seriously affects the performance of the consortium chain. Finally, the C/S architecture used in the PBFT algorithm cannot dynamically sense the number of nodes. With the increase in the number of nodes, the performance drops sharply, which is not suitable for large-scale dynamic networks, limiting the application of blockchain technology in financial services^24^, supply chain management^25^, the Internet of Things (IoT)^26–28^ and other fields. Therefore, in response to the above problems, many scholars have proposed different approaches to improve the performance and efficiency of PBFT.

Lao Laphou et al.^29^ proposed a location-based and scalable PBFT consensus algorithm. Fixed nodes have more computational power than mobile nodes, and the possibility of becoming malicious nodes is very small. This algorithm reduces the consensus overhead and ensures the security of the system by selecting a fixed and trusted node as the consensus participant, but decentralization is also greatly reduced. Zhujun et al.^30^ quantified whether the nodes are reliable based on both security and computational capability and classified the nodes into four types. Only the management nodes among each type can be included in the selection of the master nodes as alternate master nodes, while only the candidate nodes with more votes can be turned into management nodes to guarantee system security. Gan et al.^31^ proposed an improved PBFT consensus algorithm, ePBFT, which improves the master node selection method of PBFT by setting the node life cycle so that the nodes can connect and exit dynamically and by the longest chain principle. Yong et al.^32^ proposed a credit-based improved PBFT consensus algorithm (CPBFT). The original C/S architecture is changed to a P2P architecture, the consensus step is reduced, the credit coefficient is introduced, and the voting method is used to select the master node so that the probability of a node being selected as the master node is influenced by its past behavior. Experiments show that the CPBFT algorithm reduces data transmission and improves throughput compared to the PBFT algorithm. The SG-PBFT^33^ consensus algorithm optimizes the original PBFT consensus process and uses a scoring grouping mechanism to achieve higher consensus efficiency. Kai et al.^34^ improved system security by selecting master nodes through a reputation model. Riyad et al.^35^ proposed a practical Byzantine fault-tolerant algorithm S-PBFT to address the problems of high communication overhead and low efficiency of the traditional Byzantine fault-tolerant algorithm. The algorithm adds a node scoring mechanism, and all nodes are classified into consensus nodes, candidate nodes, and early nodes. To ensure that the nodes are as reliable as possible, the node scoring is dynamically changed according to the behavior of each node. EPBFT^36^ added a consensus node election based on a verifiable random function (VRF) to the original algorithm, making it more suitable for dynamic networks.

For consistency protocol improvement, Yanjun et al.^37^ proposed a high-performance and scalable Byzantine fault tolerance (HSBFT) to optimize the consistency protocol of PBFT and reduce its complexity from O(N^2^) to O(N), which improves the consensus efficiency but also weakens the polycentric characteristics of the consortium blockchain. Yuxi et al.^38^ proposed a scalable hierarchical Byzantine fault-tolerant algorithm SHBFT to form a node structure with the same characteristics, which facilitates data storage and node management^33,39^. It can reduce the size of nodes and simplify the complexity of consensus. The SG-PBFT algorithm^33^ improved the traditional PBFT consensus algorithm by optimizing the consensus process and using a scoring mechanism; the method greatly improves consistency efficiency and can effectively prevent single-node attacks. Yuhao et al.^40^ proposed a voting reward and punishment scheme and its corresponding credit evaluation scheme, which can not only motivate reliable nodes but also reduce the participation of abnormal nodes in the consensus process and establish a virtuous cycle of the system. At the same time, the scheme proposed a PBFT-based consistency and checkpointing protocol, which can improve the efficiency and flexibility of the system. Guangxia et al.^41^ and Jian et al.^42^ used a hashing algorithm to partition consistency nodes, which simplifies the consistency protocol and can avoid a large amount of communication between nodes, reduce the communication complexity of the network, and improve the scalability of the network, but cannot identify Byzantine nodes. Pengbo et al.^43^ introduced a node credit scoring mechanism and simplified the consistency protocol. The experimental results show that the CSPBFT algorithm can shorten the transaction latency and improve the long-term operational efficiency of the system. The MCPBFT^44^ algorithm divides the many nodes involved in logistics information into multiple consensus sets and improves the consistency protocol into two stages based on PBFT. The results show that the MCPBFT algorithm can effectively improve the efficiency of consensus, ensure the timely update of logistics information, and improve the practicality of the traceability model.

## Working principle and defects of PBFT

### How the PBFT algorithm works

PBFT is considered one of the best algorithms to solve the Byzantine problem^13^, which has three roles: client, master node, and replica node. When a client submits a transaction request, it is immediately sent to the master node, which initiates a transaction vote across the network, and the replica and master nodes work together to maintain the validity of the transaction vote. When the master node fails, a view change procedure is triggered to select a new master node.

**Figure 1:**
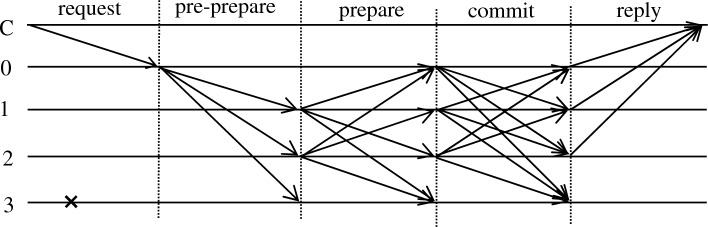
PBFT algorithm flow.

The PBFT algorithm flow is shown in Fig. 1.

First, client C sends a message m to master node 0. The master node initiates the five-segment protocol of PBFT: request, pre-prepare, prepare, commit and reply. C denotes the client node, and 0 to 3 denote the consensus node, where 0 is the master node and 3 is the fault node.

*Request stage* The client node generates a message digest and adds request operation o and timestamp t to construct the request. After completing the signature, <REQUEST, o, t, C > σ_C_ is sent to the master node.

*Pre-preparation stage* After receiving the message, the master node constructs < <PRE-PREPARE, v, n, d > σ_0_, m> and broadcasts it to the replica node, which determines whether the following is satisfied, and receives the message if it is:

- Checked that the message digest d is consistent with the digest generated by m.
- Whether v in the received message is consistent with the current view.
- Check if the same n and v but different d messages are received locally.

*Preparation phase* The replica node receives the pre-preparation message from the node and enters the preparation phase by broadcasting the message <PREPARE, v, n, d, i > σ_i_ to the other nodes. The node receives the preparation message to verify that the following requirements are met and receives 3f+1 (including itself) messages to enter the commit phase:

- Prepare for whether the message signature is correct.
- Determine whether the current node received messages with the same v and n but different signatures.
- Determine the summary generated by the current node and whether it is consistent with d.

*Commit phase* The commit phase requires broadcasting < COMMIT, v, n, i > σ_i_, and the other nodes determine:

- The received message signature is correct.
- The current node does not receive the same n under a v.
- The current node generates the same message summary as the received d.

*Reply phase* After the current node receives 2f + 1 (including itself) commits, it records the message to the local log and replies to the client.

After client C receives the reply, the entire network reaches a consensus, and the message is committed to the local database.

### Deficiencies of the PBFT algorithm

Despite the obvious advantages of PBFT over the other consensus algorithms, the mechanism still struggles with the following problems.

1. Master node election is too arbitrary. In the existing PBFT algorithm, the master node election tends to take a rotating or random approach among all nodes, which makes the selection of master nodes vulnerable to DDoS attacks and sybil attacks in P2P networks with high security risks.
2. The voting process is too complex, and the three-stage protocol broadcast process of the PBFT consensus algorithm requires network-wide forwarding with great communication overhead, which imposes an excessive load on the consensus network.
3. Nodes in the PBFT consensus algorithm cannot join and exit at will, affecting system availability.
4. The nodes lack an effective reward and punishment mechanism, which cannot reward honest nodes or punish evil nodes, making the nodes less loyal and motivated.

## CBFT Consensus algorithm

### Overall thinking

To improve the problems of the above PBFT algorithm, a consensus algorithm CBFT based on grouping and credit model improvement is proposed. The main improvements of the CBFT mechanism include the following aspects.

1. A grouping strategy is proposed. A large number of nodes are divided into multiple groups to reduce the size of the nodes, the complexity of the consensus is simplified and the system overhead is reduced.
2. A credit model is defined to optimally manage node elections. Based on data such as the response performance of nodes, historical credit value and the correct rate of reaching consensus on transactions, the trustworthiness of nodes is evaluated, and nodes with high credit value are selected as management nodes, which can effectively reduce the probability of management nodes being Byzantine nodes and improve system security.
3. Optimize the consistency protocol. The three-stage main protocol of out-group PBFT is optimized into two stages to improve consensus efficiency.

**Figure 2:**
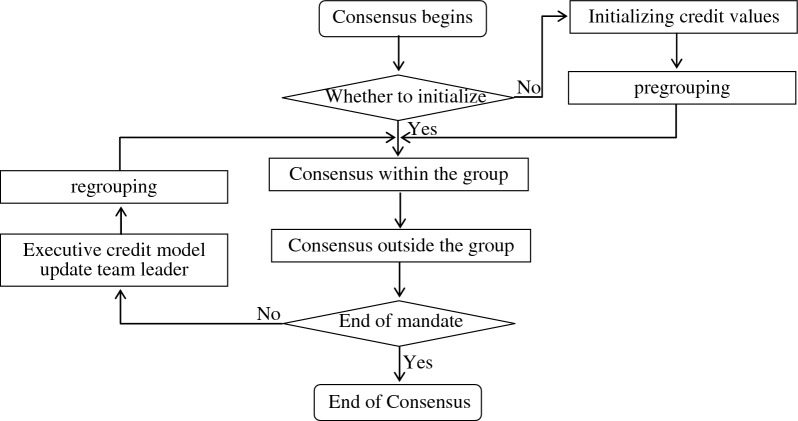
CBFT consensus process.

The consensus process is shown in Fig. 2.

### Grouping strategy

Using the authentication mechanism for nodes to join the consortium chain, m nodes are randomly selected as the initial management nodes, and the nodes are divided into G consensus sets based on the response rate of the remaining nodes to the management nodes as a grouping basis.

Management node i examines the list of members in group G_i_, determines whether the number of nodes is greater than N_max_, stops broadcasting the message, is less than N_max_, and broadcasts the receiving member message <GROUP, t_1_, G_i _> σ_i_, where N_max_ is the maximum number of nodes allowed in the group. t_1_ is the timer, G_i_ is the list of nodes contained in the current group, and σ_i_ is the signature of management node i.

Node x receives a broadcast message from the management node, verifies that the message signature is correct, and if it is correct, initiates a group entry request to the management node <GROUP-REQUEST,x,t_2_>σ_x_.

The management node receives the group admission application, verifies that it is correct, adds node x to the group membership list G_i_ and sends it a message to confirm <GROUP-COMMIT, t_3_, G_i _> σ_i_.

At the end of grouping, the management node broadcasts the respective group list G_i_, and if it is verified to be correct, it sends this information down to the members in each group to complete the group confirmation.

The management node is the node with the highest credit value in the group and participates in the global consensus as the proxy node of the group. Therefore, after one round of consensus, the node credit value is updated, a new management node is selected based on the new credit value of the node, and the group is regrouped according to the above process.

**Figure 3:**
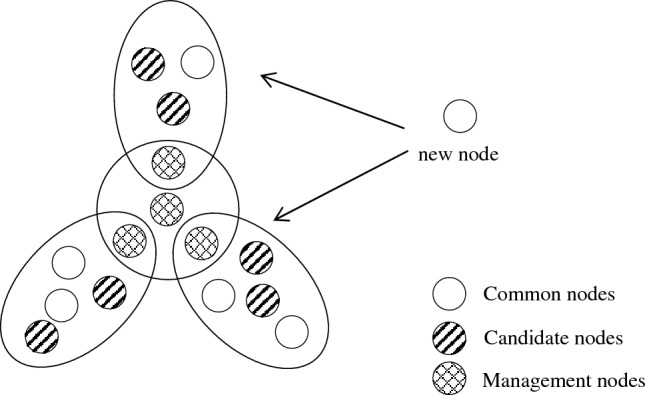
New node entry process.

To address the problem that nodes cannot be dynamically connected in PBFT, CBFT establishes a node entry and an exit mechanism, which enables authorized nodes to dynamically join the consensus network without affecting the system structure, as shown in Fig. 3.

When a new node wants to join the network, it first looks for the nearest management node and sends a search request to the surrounding nodes. The node forwards information from the management node to the new node with a timestamp, compares the timestamps, and sends a group request to the management node of the node that received the reply first. The management node adds the new node information to the list of group members and provides the new node with information about the other nodes in the group, and the new node joins the network completion. The new node enters the network as a common node, does not participate in the consensus, and only receives the consensus results.

In the node exit mechanism, if the exit from the network is a management node, it first broadcasts a message to downgrade the status to a candidate node, waits for the successful election of a new management node, and reports the exit to the management node. If it is a candidate node or a common node to exit the network, it can directly apply to the management node.

### Credit model

PBFT determines the master nodes sequentially according to the Formula p = v mod N. The anomalous nodes are likely to be elected as master nodes, which affect the security of the system. In the improved CBFT algorithm, the credit model is introduced to optimize the master node election so that the node with a high credit value has a higher probability of becoming the master node.

In the credit model, the node credit value is set to [0,100] with an initial value of 40, and the nodes involved in the CBFT consensus are divided into three categories according to the size of the credit value: management nodes, candidate nodes, and common nodes. Both management nodes and candidate nodes are consensus nodes, and common nodes only receive the consensus results and do not participate in the consensus process, as shown in Table 1.

**Table 1:** Node credit value categories.

Credit value	[0–40]	(40–60)	[60–100]
Node category	Common nodes	Candidate nodes	Management nodes

To evaluate the current credit value of the node, the node trustworthiness is measured using data such as the node's responsiveness performance, historical credit value, and correctness of reaching transaction consensus as metrics.

Definition 1: Latency index is the delay in the process of responding to various messages, expressed as

1 $${\text{D}}\left( i \right) = \left[ {1 - \left( {\frac{{{\text{d}}_{{i{\text{j}}}} }}{{d_{\max } }}} \right)^{3} } \right]*100$$

where d_ij_ denotes the delay of the jth transaction of node i; d_max_ denotes the maximum delay allowed by the exchange, and if the maximum delay is exceeded, it indicates that the node failed to execute the transaction.

Definition 2: Transaction completion rate with a penalty mechanism refers to the percentage of nodes that successfully participate in each transaction after entering the network, and is expressed as

2 $${\text{T}}\left( i \right) = \frac{100}{{\text{n}}}\sum\limits_{i = 1}^{m} {\mu_{i} }$$

where n is the total number of system transactions and m denotes the number of transactions completed by node i. μ is the identifier of whether the transaction is successful or not, with a successful transaction μ of 1 and a failed transaction μ of -1. Both the promotion effect of successfully completed transactions on the node and the adverse effect on the node by affecting the normal conduct of transactions are considered, which can better distinguish the credit value of the node.

Definition 3: Influence of historical credit values. The credit status of the current node is influenced by the historical credit value, and is expressed as

3 $${\text{C}}\left( i \right)^{\prime } = z{\text{C}}\left( {i - 1} \right)$$

The coefficient z indicates the degree of historical state influence.

Definition 4: The final credit score of a node is calculated as follows.

4 $$\begin{gathered} {\text{C}}\left( i \right) = \frac{1}{3}\left( {xD\left( i \right) + yT\left( i \right) + {\text{C}}\left( i \right)^{\prime } } \right) \hfill \\ \, = \frac{1}{3}\left( {100x\left[ {1 - \left( {\frac{{{\text{d}}_{{i{\text{j}}}} }}{{d_{\max } }}} \right)^{3} } \right] + 100y\left( {\frac{1}{{\text{n}}}\sum\limits_{i = 1}^{m} {\mu_{i} } } \right) + z{\text{C}}\left( {i - 1} \right)} \right) \hfill \\ \end{gathered}$$

x is the weight of the node's transaction latency, y is the weight of the node's own completed transactions, and x + y + z = 1. The credit model intuitively reflects the node's performance in the consensus. If a node has small latency, a high transaction completion rate, and good historical credit value, it is highly credible; conversely, if a node has large latency, a low transaction completion rate, and poor historical credit value, it is less credible.

### Optimized consistency protocol

**Figure 4:**
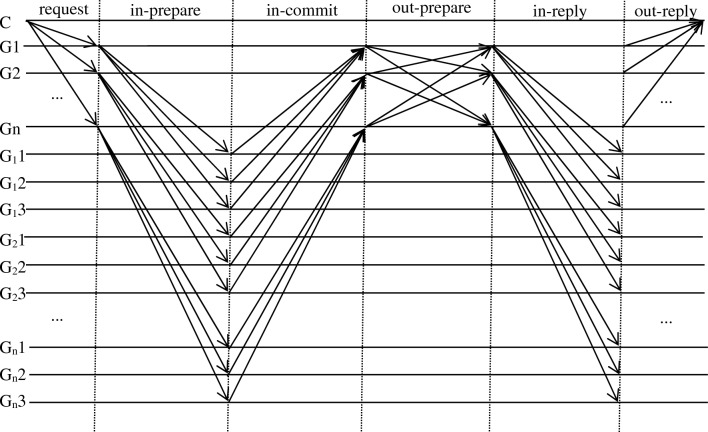
CBFT improved the consensus process.

The CBFT consensus algorithm contains in-group and out-group consensus, which mainly achieves consensus among the nodes involved in consensus. The group member nodes receive the management node broadcast, and in the intragroup preparation and commit phases, the management nodes receive enough information about the proposal. When the out-group preparation phase is completed, it means that enough nodes have verified and passed the proposal initiated by the client, and the out-group commit phase is only the confirmation of the proposal passed in the preparation phase to ensure that enough nodes have completed the proposal verification. Thus, the three-phase protocol outside the group can be optimized into two phases, as shown in Fig. 4. Nodes join the network, randomly elect the initial management node, form different groupings according to the grouping policy, initialize the credit values of each node, and execute the optimal consistency protocol of the CBFT algorithm, as follows.

- *Out-group pre-preparation phase* upon receiving a request from a client, the management node validates and sequences the request and broadcasts a pre-preparation message < OUT-PRE-PREPARE, n, t, v, D(m) > σ_i_ to the group members, where n is the sequence number assigned to the request, t is the timestamp, v denotes the view number, D(m) is the message digest, and σ_i_ is the signature of management node i.
- *Intragroup preparation phase* group members receive and validate the pre-preparation message, at which point all nodes in the network have received the pre-preparation message.
- *Intragroup commit phase* group members verify the proposal, and if it is correct, the response is sent to the proposal < IN-COMMIT, n, t, v, D(m) > σ_x_ to the group leader, where σ_x_ is the signature of node x.
- *Out-group preparation phase* the management node receives responses from a sufficient number of group members, turns on global consensus, and broadcasts the result of the in-group consensus < OUT-PREPARE, n, t, v, D(m) > σ_i_ to all management nodes.
- *Intragroup reply phase* the management node receives more than 2f. identical messages and sends a request acknowledgment < IN-REPLY, n, t, v, D(m) > σ_i_ to the group members.
- *Out-group reply phase* the management node replies to the client < OUT-REPLY, n, t, v, D(m) > σ_i_, and when the client receives f + 1 identical reply messages, the message is added to the end of the blockchain and consensus ends.

At the end of one round of consensus, the credit value of each node is calculated according to the credit model, the group reelects the management node, and repeats the above operation until all transactions are completed.

## Experiments and analysis of results

### Experimental environment

A blockchain system is simulated and implemented based on the Java programming language, and for comparison and reference, the PBFT and GPBFT^45^ algorithms are used to jointly demonstrate the superiority of this scheme. After the system runs for 5 minutes, the client initiates 200 sets of requests and selects different numbers of nodes into 5 consensus sets, each with the same initial trust value50, to test the performance of three aspects of throughput, latency, communication overhead, and security in the same network environment. Five hundred trials are conducted for each set, and the average of 500 trials is taken as the test result.

### Throughput

**Figure 5:**
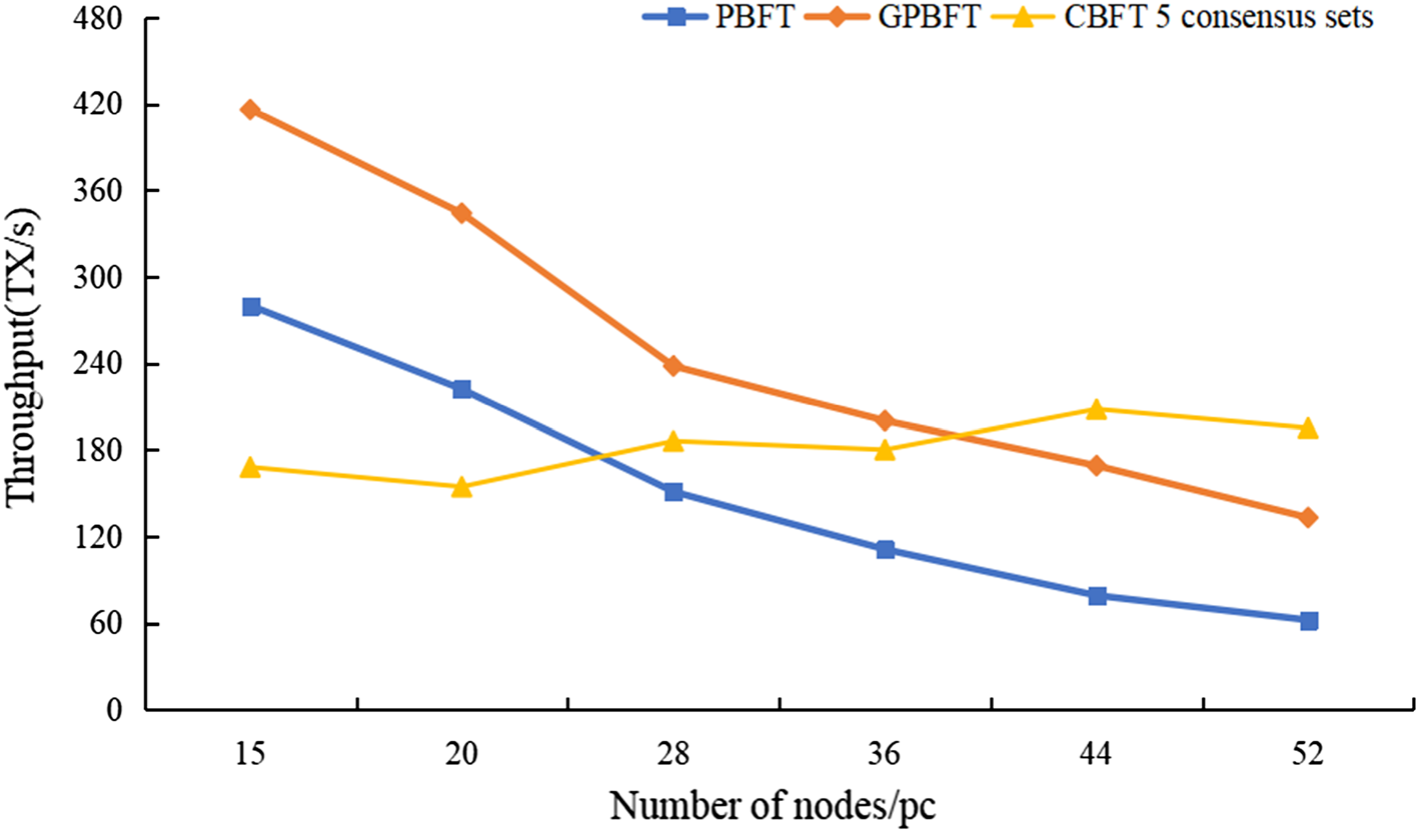
PBFT and CBFT throughput comparison.

In blockchain systems, the throughput refers to the number of transactions processed by the system per unit of time, usually expressed as TPS. The higher the throughput is, the better the system's ability to process transactions, which is an important indicator of the system's concurrent processability. The calculation formula is as follows:

5 $${\text{TPS}} = \frac{{{\text{Transactions}}_{{\Delta {\text{t}}}} }}{\Delta t}$$

where Transactions_Δt_ denotes the number of transactions processed in time Δt. As shown in Fig. 5, the throughput of CBFT is lower than that of the traditional PBFT and GPBFT consensus algorithms when the number of nodes is small. After the number of nodes exceeds 40, the TPS of the PBFT and GPBFT algorithms continues to decline, while CBFT still maintains a more stable throughput. The main reason for this is that the number of nodes is small, and the preliminary grouping will generate larger resource consumption. When the number of nodes exceeds a certain number, the throughput of CBFT will exceed that of PBFT and GPBFT. Therefore, as the number of nodes increases, CBFT has more obvious advantages and is suitable for large consortium chains.

### Consensus latency

Consensus latency is the time elapsed from the beginning of a transaction to the end of the transaction and is an important indicator of how fast the consensus algorithm is running; a low consensus latency allows transactions to be confirmed quickly, making the system more secure and practical. The formula is expressed as follows:

6 $${\text{T}}_{{\text{d}}} = T_{{\text{c}}} - T_{r}$$

**Figure 6:**
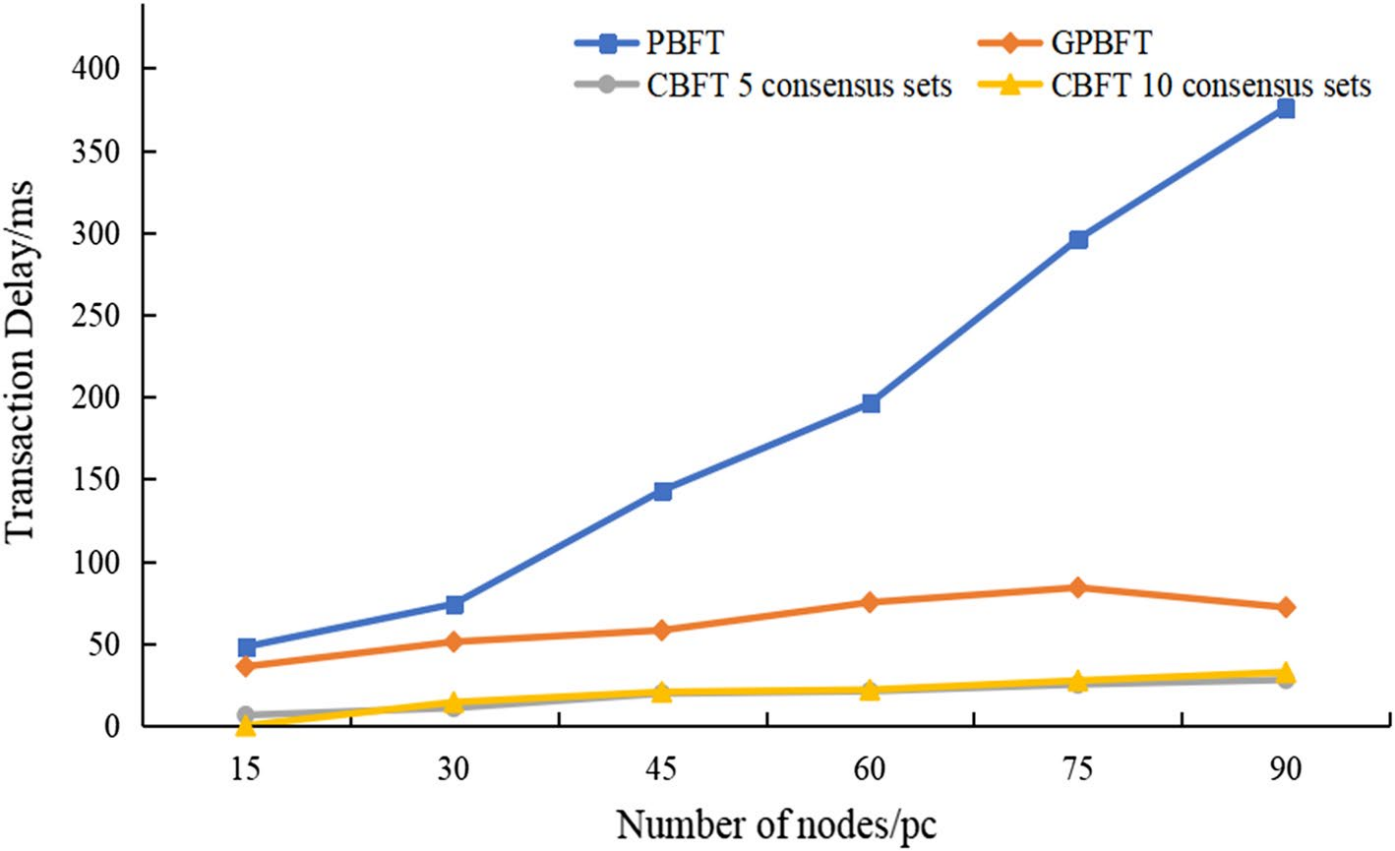
Comparison of consensus latency of PBFT and CBFT.

T_c_ denotes the transaction confirmation time, and T_r_ denotes the transaction generation time. In Fig. 6, it can be seen that the consensus latency increases gradually with the increase in the number of nodes, but the consensus latency of CBFT is considerably lower than that of GPBFT and PBFT; with the increase of the number of nodes, the latency growth rate of PBFT is substantially larger than that of GPBFT and CBFT; especially when the number of nodes exceeds 60, the latency of PBFT has reached more than 200 ms, and that of GPBFT reaches 75 ms, while the delay of CBFT still remains approximately 20 ms. The results show that CBFT makes a great improvement in terms of latency.

### Communications overhead

The communication overhead is the amount of communication incurred by the nodes in the system to execute the consensus algorithm.

Assuming that the number of nodes in each consensus set is the same (not less than 3), the number of consensus sets should be no less than 4, and the total number of nodes in the system is N (N>12). There are three phases in PBFT where messages need to be sent for communication. First, the client sends the request to the master node, which sends the pre-preparation message to all replica nodes, and the number of communications in the pre-preparation phase is (N−1). After receiving the message, the node verifies and sends the message in the preparation phase, and the number of communications in this phase is (N−1)^2^. Finally, in the commit phase, the node receives the preparation message, and when the verification result is consistent, the submitted message is sent to all nodes, and the number of communications in this phase is N(N−1). Based on the communication time of the above three phases, we simplify PBFT to complete the consistent communication time T_1_ as follows:

7 $${\text{T}}_{1} = {\text{N}} - 1 + ({\text{N}} - 1)^{2} + {\text{N}}({\text{N}} - 1) = 2{\text{N}}({\text{N}} - 1)$$

In CBFT, there are two phases in each consensus set where messages need to be sent for communication. Assuming that each consensus set has M(M ≥ 3) nodes, the number of communications in the preparation phase is N-N/M and the number of communications in the commit phase is N-N/M. Thus, the total number of communications in the consensus set T_2_ is:

8 $${\text{T}}_{{2}} = {\text{N}} - \frac{{\text{N}}}{{\text{M}}} + {\text{N}} - \frac{{\text{N}}}{{\text{M}}} = 2\left( {{\text{N}} - \frac{{\text{N}}}{{\text{M}}}} \right)$$

The consensus within the consensus set, the participation in global consensus, and the number of communications T_3_ for:

9 $${\text{T}}_{{3}} = \frac{{\text{N}}}{{\text{M}}}\left( {\frac{{\text{N}}}{{\text{M}}} - 1} \right) = \left( {\frac{{\text{N}}}{{\text{M}}}} \right)^{2} - \frac{{\text{N}}}{{\text{M}}}$$

In summary, the total number of CBFT communications T_4_ is:

10 $${\text{T}}_{{4}} = {\text{T}}_{{2}} + {\text{T}}_{{3}} = \left( {\frac{{\text{N}}}{{\text{M}}}} \right)^{2} - \frac{{{\text{3N}}}}{{\text{M}}} + 2{\text{N}}$$

When N > 12 and T_4_ < T_1_, the improved communication overhead is smaller.

**Figure 7:**
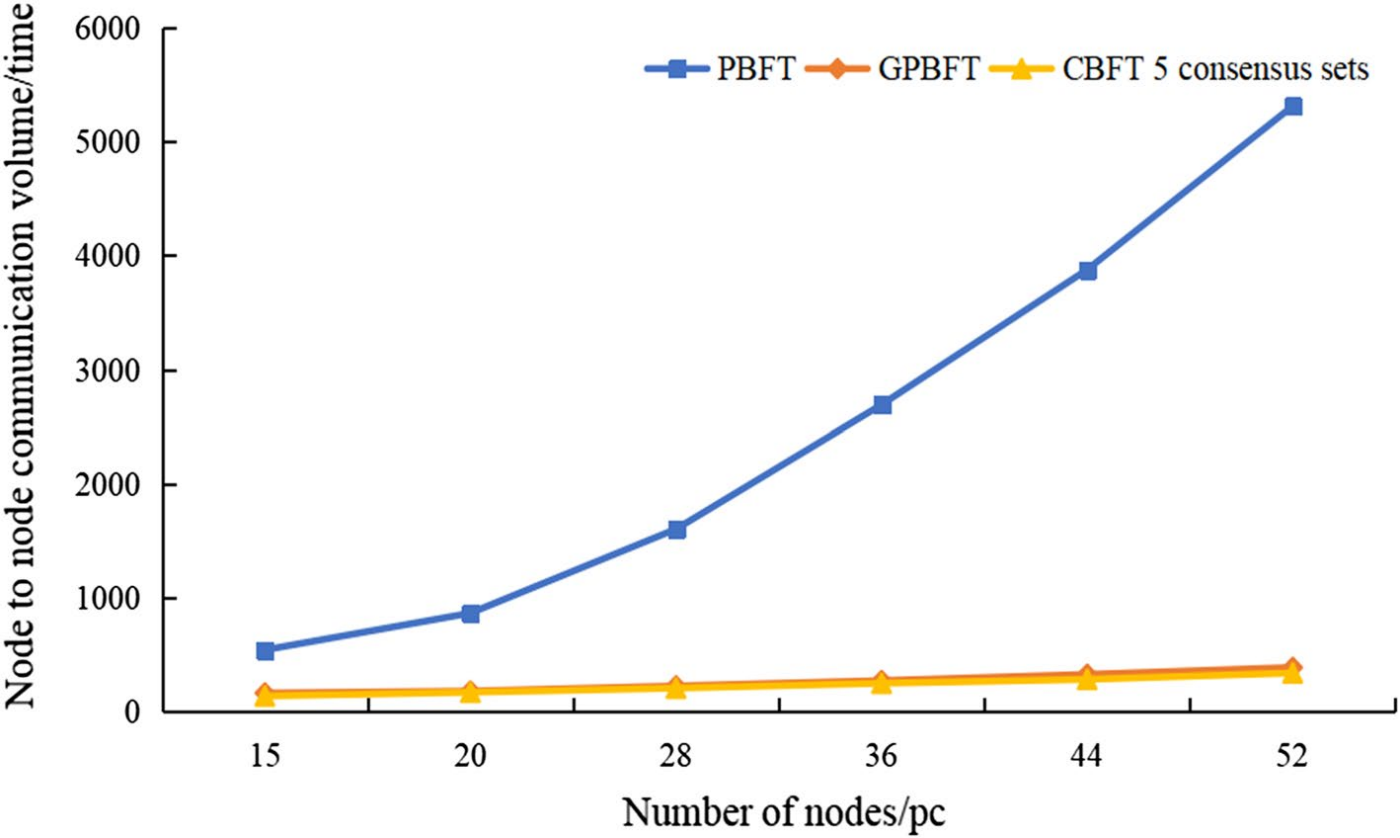
Comparison of PBFT and CBFT traffic.

Figure 7 shows a comparison of the communication overhead of the CBFT, GPBFT, and PBFT consensus algorithms. It can be seen that the communication overhead of GPBFT and CBFT is much smaller and grows slowly with the increase in the number of consensus nodes in the whole blockchain network. When the number of network nodes is 36, the communication overhead of PBFT is 2691, GPBFT is 271, and CBFT is 248 (5 consensus sets), which is 90.8% lower than PBFT and 8.5% lower than GPBFT. The main reason for these results is that CBFT divides the nodes in the system into G consensus sets, and only the management nodes within the consensus sets participate in the consensus outside the consensus sets, thus reducing the number of nodes involved in the global consensus and reducing the amount of information sent between nodes to each other.

### Security testing

Security is an important attribute in blockchain systems, and malicious nodes are an important cause of consensus failure. In this paper, we introduce a credit model to calculate the credit value of nodes and use this credit value as the basis for selecting master nodes, which can effectively reduce the probability of malicious nodes becoming management nodes and improve system security. In this section, we analyze the security of CBFT and discuss it in terms of two aspects: the throughput under different Byzantine node occupancy ratios and the maximum number of Byzantine nodes that the algorithm can tolerate.

### Throughput under different Byzantine occupancy ratios

**Figure 8:**
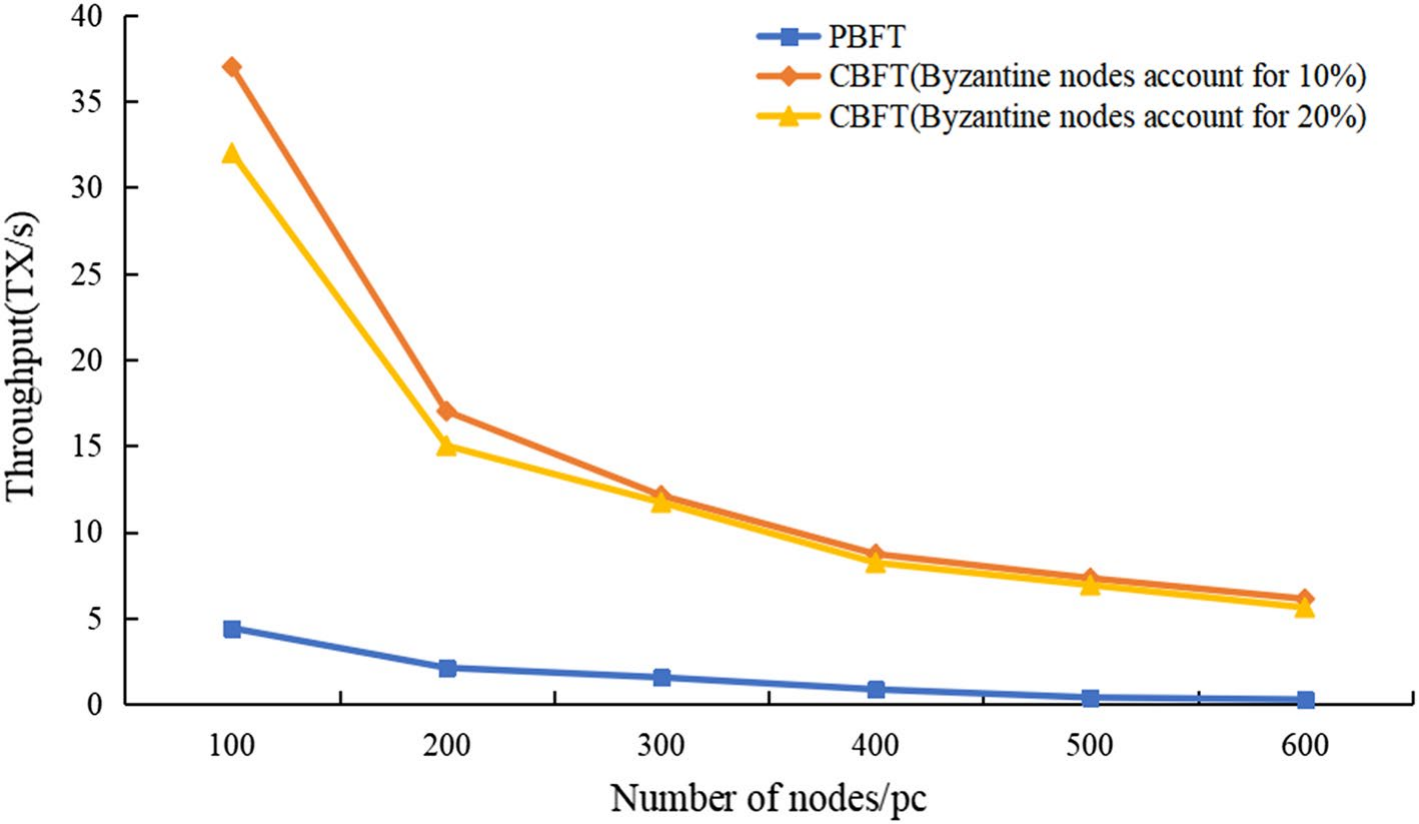
Throughput of the PBFT and CBFT algorithms with different Byzantine occupancy ratios.

To test the performance of CBFT under various numbers of nodes and different Byzantine node occupancy ratios, we tested the throughput of CBFT at 100, 200, 300, 400, 500, and 600 nodes and set the ratio of malicious nodes to 10 and 20%. The results of their experimental comparisons are shown in Fig. 8.

In the figure, we can see that the throughput of the network gradually decreases as the number of nodes increases, and the throughput of CBFT is considerably better than that of PBFT, while we find that the throughput does not increase substantially after the percentage of Byzantine nodes increases. The main reason for this is that Byzantine nodes are dispersed within each consensus set due to the grouping strategy, and they cannot be elected as management nodes due to the credit model, which cannot further disrupt the global consensus. Even if there is such an unexpected situation in one consensus set, the other consensus sets can complete the consensus process because of the fault tolerance of the global PBFT consensus.

### The maximum number of Byzantine nodes that can be tolerated

**Figure 9:**
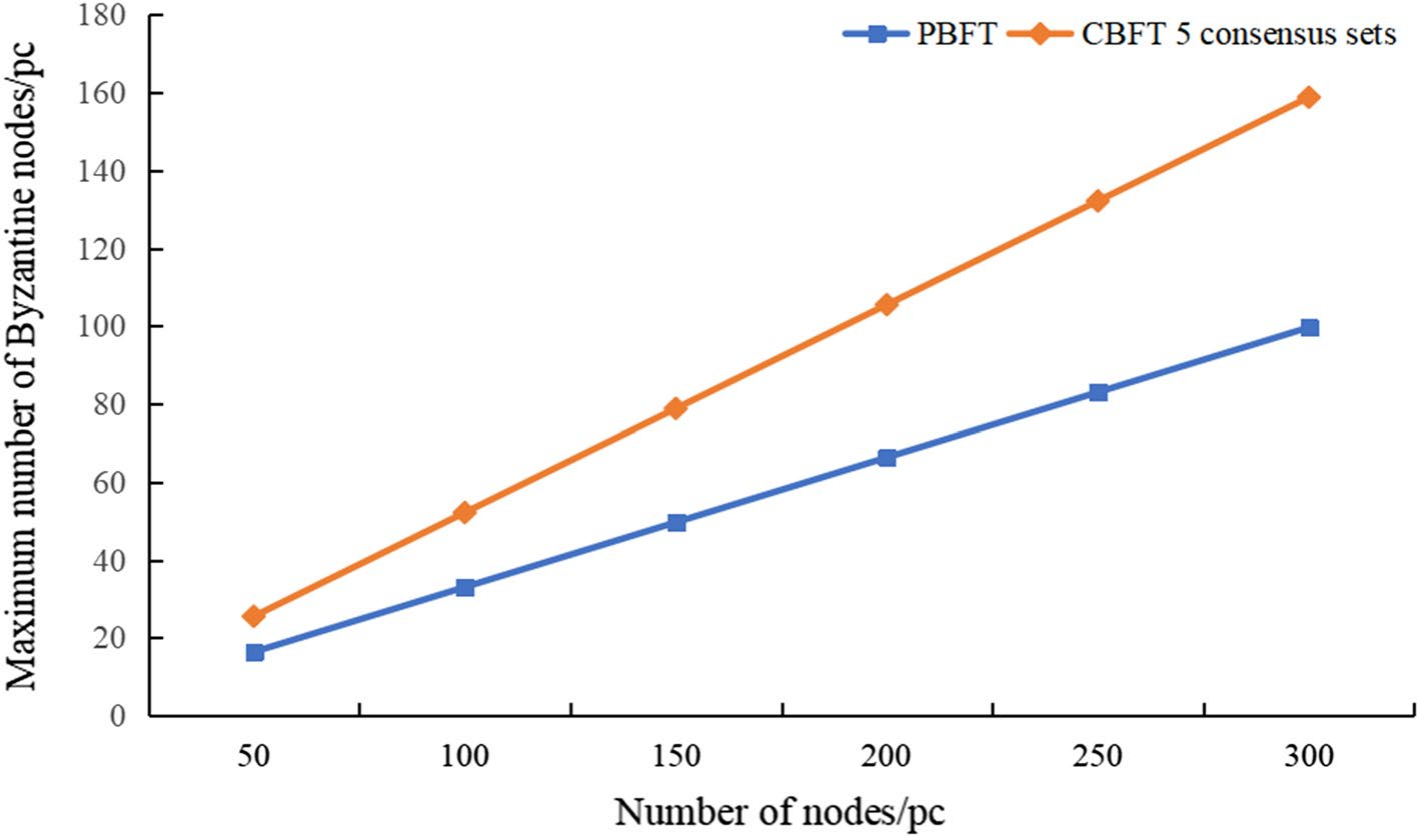
Maximum number of Byzantine nodes tolerated by the PBFT and CBFT algorithms.

The maximum number of Byzantine nodes that can be tolerated by PBFT is 1/3, and PBFT fails when it exceeds 1/3. According to the grouping strategy of CBFT, CBFT can tolerate 1/3 of the consensus set to be all Byzantine nodes, and the rest of the consensus set can tolerate at most 1/2 of the nodes to be Byzantine nodes; therefore, the fault tolerance of the CBFT algorithm is considerably higher than that of the PBFT algorithm. Figure 9 shows the maximum number of Byzantine nodes tolerated by the PBFT and CBFT algorithms with different numbers of nodes. The fault tolerance of CBFT is higher than that of PBFT and increases with increasing node size.

## Conclusion

To address the problems of limited network scale supported by the PBFT algorithm and unfavorable to the development of large consortium chains, an improved Byzantine fault-tolerant algorithm (CBFT) based on grouping and credit grading is proposed to divide large-scale network nodes into different consensus sets and select consensus nodes based on credit values. Simulation results show that compared with the PBFT and GPBFT algorithms, CBFT has considerably improved the system performance and reliability in terms of throughput, latency, communication overhead, and security. However, some limitations need to be overcome. For example, the identity of nodes joining the network cannot be verified at present and can only be authenticated by the MSP of the consortium chain. In future work, we will investigate how to perform node identity authentication to further improve system security. Moreover, we intend to explore the possibility of applying blockchain to smart agriculture to further improve the algorithm and promote blockchain development (Supplementary material).

### Supplementary Information

Supplementary Information.

## Data Availability

The datasets used and/or analysed during the current study available from the corresponding author on reasonable request.
